# Dynamic light scattering imaging

**DOI:** 10.1126/sciadv.abc4628

**Published:** 2020-11-06

**Authors:** Dmitry D. Postnov, Jianbo Tang, Sefik Evren Erdener, Kıvılcım Kılıç, David A. Boas

**Affiliations:** 1 Neurophotonics Center, Boston University, Boston, MA 02215, USA.; 2 Biomedical Sciences Institute, Faculty of Health and Medical Sciences, Copenhagen University, Copenhagen 2200, Denmark.

## Abstract

We introduce dynamic light scattering imaging (DLSI) to enable the wide-field measurement of the speckle temporal intensity autocorrelation function. DLSI uses the full temporal sampling of speckle fluctuations and a comprehensive model to identify the dynamic scattering regime and obtain a quantitative image of the scatterer dynamics. It reveals errors in the traditional theory of laser Doppler flowmetry and laser speckle contrast imaging and provides guidance on the best model to use in cerebral blood flow imaging.

## Teaser

Nature of red blood cell dynamics and light scattering interactions unraveled to accurately probe cerebral blood flow.

## INTRODUCTION

The question of the appropriate model to use to interpret laser speckle fluctuations has been debated for decades, especially in laser Doppler flowmetry (LDF) and laser speckle contrast imaging (LSCI) blood flow measurement applications (*1*–*6*). The model is defined by the form of the intensity autocorrelation function *g*_2_(τ), which is related to the field temporal autocorrelation function *g*_1_(τ). The latter can be quantitatively related to the dynamics of the light scattering particles including flowing red blood cells (*7*, *8*). The relation between *g*_2_(τ) and *g*_1_(τ) depends on the amount of static scattering present in the sample (*9*–*13*), measurement-specific parameters related to source coherence (*14*, *15*), detector speckle averaging (*16*) and detector noise (*9*, *17*, *18*). The form of *g*_1_(τ) depends on the amount of light scattering (i.e., single or multiple scattering) and the type of particle motion (i.e., ordered or unordered) (*3*, *7*, *19*). Although these forms of the field correlation functions have been established for over 30 years, there is no agreement nor experimental support on what scattering and motion regimes are relevant for the varied biomedical applications.

Experimental approaches and processing schemes have been suggested to address measurement noise (*9*, *17*) and static scattering (*10*, *11*, *13*, *20*) as well as to investigate the form of the field correlation function (*3*, *10*). The multiexposure speckle imaging (MESI) technique (*9*, *10*, *21*) has been introduced to probe the temporal dynamics of the speckles indirectly by changing the exposure time to measure the impact on speckle contrast. MESI was first used to quantify the impact of static scattering to improve estimates of relative blood flow changes (*9*) but was not able to resolve questions regarding the form of the field correlation function due to insufficient temporal sampling to resolve intensity fluctuations directly.

Using a high-speed camera and recording back-scattered laser light at more than 20,000 frames/s, we introduce the first wide-field dynamic light scattering imaging (DLSI) for in vivo biomedical applications. It combines (i) the ability to resolve the temporal speckle intensity fluctuations and directly measure *g*_2_(τ), as in dynamic light scattering (*22*, *23*) and diffuse correlation spectroscopy (*24*, *25*) techniques, with (ii) high-resolution (limited only by the objective) wide-field imaging, typical for LSCI and MESI. DLSI permits estimation of the best-fitting light scattering model directly for every pixel individually, resulting in a high-resolution quantitative image of the dynamics and scattering properties of the particles in the sample. It allows us to solve the problem of how to quantitatively interpret data measured by methods in which *g*_1_(τ) is assumed beforehand, including LSCI (*8*, *18*, *26*), MESI (*9*, *10*) and LDF (*7*, *27*, *28*).

## RESULTS

### Laser speckle intensity temporal autocorrelation function

**Fig. 1:**
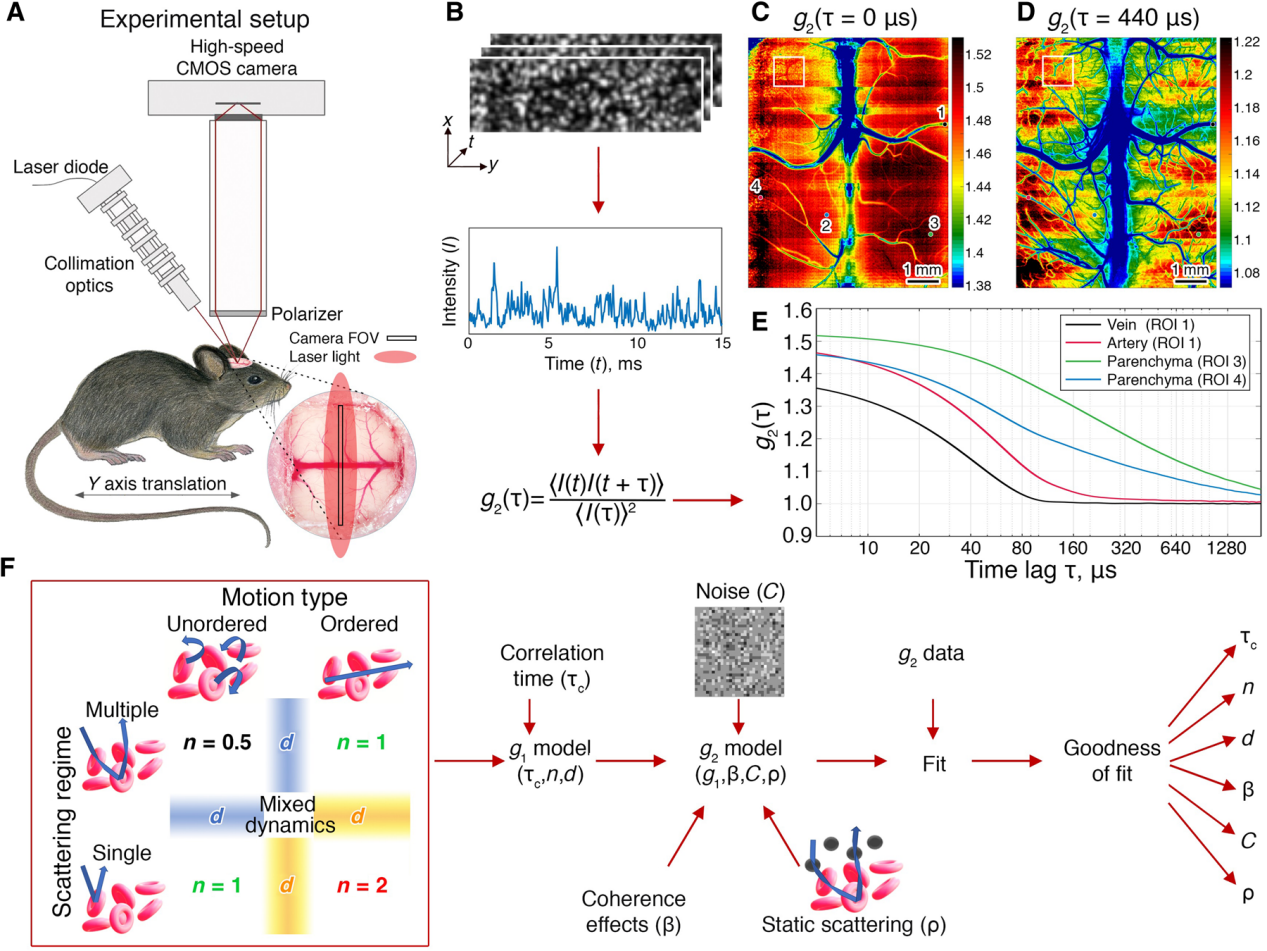
Dynamic light scattering imaging. (**A**) Diagram of the imaging system. (**B**) Data flow and *g*_2_(τ) calculation. (**C** and **D**) Spatial maps of *g*_2_(τ) at the specific time lags of τ = 0 and 440 μs. (**E**) The *g*_2_(τ) at selected 3 × 3 regions of interest that belong to a vein and artery (approximately 150 and 70 μm in diameter, respectively), and two regions in the parenchyma as indicated in (C). Notice that at τ = 0, some of the vessels appear to have higher values compared with the surrounding parenchymal tissue [white box in (C)]. At later τ, the relationship is reversed with lower values of *g*_2_(τ) observed in the vessels [see the white box in (D)]. (**F**) Flowchart of the model and fitting process. Red box on the left describes how the *g*_1_(τ) form depends on the motion type and scattering regime.

We apply DLSI to measure the intensity autocorrelation function *g*_2_(τ) in the mouse brain (Fig. 1, A and B; online methods). Examples of the calculated images of *g*_2_(τ = 0 μs) and *g*_2_(τ = 440 μs) and the measured *g*_2_(τ) for different regions of interest are shown in Fig. 1 respectively). The *g*_2_(τ = 440 μs) shows an example of the intensity autocorrelation function being fully decorrelated for most of the vessels, but not for the parenchymal regions. For pixels belonging to parenchymal regions, the correlation function decays slower compared with that from the pixels of larger surface vessels, which, in turn, decays slower for the smaller vessels than for the large vessels as the larger vessels generally have faster blood flow (consequently one can expect faster decay for arteries than the veins, even if the vessel diameter is the same). Figure 1C shows that *g*_2_(τ = 0 μs) is not spatially uniform, that is, it varies from location to location with (i) larger vessels having a reduced *g*_2_(τ = 0 μs) value and (ii) some of the small vessels having *g*_2_(τ = 0 μs) values larger than the surrounding parenchyma (see fig. S1). The latter can be explained by more static scattering in the parenchyma, which leads to a decrease in the *g*_2_(τ = 0 ms).

To quantify characteristics of the intensity temporal autocorrelation function, we developed a comprehensive model that accounts for the dynamics of the scattering particles and the parameters of the light scattering in the sample (Fig. 1F). For the details on the derivation process, please see Methods. Briefly, we start with the Siegert relation, addressing the different forms of the field correlation function and the effects of the spatial and temporal speckle averaging (fig. S2). Then, we increase the model complexity step by step by (i) introducing a constant offset to consider the contribution of noise, (ii) accounting for the contribution from static scattering, and (iii) allowing for a mixture of forms for the field correlation function as detected light may have scattered from ordered and unordered dynamic particles either once or multiple times. For each of the complexity levels, we identify the best-fitting form of the field correlation function and then use the *F* test to compare the performance of all models (fig. S3). The final DLSI model, which provides the best fit overall and includes all of the mentioned parameters, is

$$\begin{array}{cc} g_{2}(\tau )= & 1+\beta \rho^{2}{\left(d|g_{1}^{n=X}(\tau )|+(1-d)|g_{1}^{n=1}(\tau )|\right)}^{2}+\\ & 2\sqrt{\beta }(1-\rho )\rho (d|g_{1}^{n=X}(\tau )|+(1-d)|g_{1}^{n=1}(\tau )|)+C \end{array}$$ (1)

where $g_{1}^{n}(\tau )=\text{exp}(-{\left(\tau /\tau_{c}\right)}^{n})$, τ_c_ is the decorrelation time constant, β reflects the effects of the source coherence properties and the spatial and temporal averaging of the speckle dynamics, ρ represents the fraction of the dynamic scattering component, (1 − ρ) represents the fraction of the static scattering component, and *C* is an offset caused by measurement noise. *X* depends on the second type of dynamics with a value of 0.5 for multiple scattering from unordered motion (MU_*n* = 0.5_) (*3*, *29*) or 2 for single scattering from ordered motion (SO_*n* = 2_) (*3*, *19*). *d* represents the influence of the *n* = *X* component compared with the single scattering from unordered or multiple scattering from ordered dynamics (SU/MO_*n* = 1_) (*3*, *8*).

### Dynamic scattering regimes

**Fig. 2:**
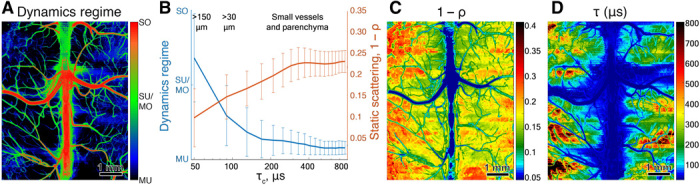
DLSI of a mouse brain cortex. (**A**) Map of the dynamic light scattering regimes identified in the mouse brain. (**B**) Variation of the dynamic regime and static scattering for different correlation times τ_c_, which, generally, is shorter for larger vessels. Regions within 50 pixels from the superior sagittal sinus were excluded from the plot. The estimated parameters with respect to the average vessel diameter are shown in fig. S4. (**C**) Map of the static scattering component 1 − ρ. (**D**) Map of the correlation time τ_c_.

Applying the model to the *g*_2_(τ) measured from the mouse brain, we obtained the correlation time τ_c_, static scattering estimate 1 − ρ, and the dynamic scattering regime, which ranges from MU_*n* = 0.5_ to SU/MO_*n* = 1_ and from SU/MO_*n* = 1_ to *SO*_*n* = 2_. Historically, SU/MO_*n* = 1_ has been assumed in LSCI (*8*, *18*) and MESI (*9*, *10*), while for LDF, either SU/MO_*n* = 1_ or SO_*n* = 2_ was used (*7*, *28*, *30*). DLSI, however, shows that neither is correct per se (see Fig. 2. The generally accepted SU/MO_*n* = 1_ regime is present only in vessels with diameters in the range of ≈30 to 150 μm, larger vessels tend to demonstrate SO_*n* = 2_ behavior, and, contrary to expectations, we found that flow in the parenchyma and small visible vessels mostly displays the MU_*n* = 0.5_ dynamics (see fig.S4 for details on how these parameters vary with respect to the average diameter). Moreover, some of the regions are best fit with the ˮmixedˮ dynamics model [fig. S3, (i)]. SO_*n* = 2_ dynamics that occur in the center of the largest vessels transition to SU/MO_*n* = 1_ dynamics on the edges of these vessels, which is likely single scattering from unordered motion of red blood cells resulting from shear induced diffusion of the red blood cells (*31*). In smaller connected vessels and parenchyma, we observe mixed MU_*n* = 0.5_ to SU/MO_*n* = 1_ dynamics. This can potentially be explained by the SU_*n* = 1_ dynamics becoming slow enough that the contribution of the MU_*n* = 0.5_ dynamics from red blood cells in the capillaries becomes competitive. One can expect that mixed dynamics (MU_*n* = 0.5_ to SU/MO_*n* = 1_) and MU_*n* = 0.5_ regimes are not limited to the brain and will be dominating speckle decorrelation for all tissues with a large number of small vessels, such as skin (see fig. S5) or kidney. This result has a major effect on the accurate interpretation of LSCI, LDF, and MESI measurements.

DLSI also permits estimation of the contribution of static scattering, which otherwise confounds interpretation of LSCI (*9*, *18*). From Fig. 2 we see that static scattering in the parenchyma is around 0.15 to 0.3, in agreement with MESI estimations (*10*), meaning that approximately 80% of the detected photons have experienced at least one dynamic scattering event. This is an indication that the detected light is experiencing multiple scattering events within the tissue as the cerebral blood volume represents no more than 4% of the tissue volume. This result supports recent Monte Carlo simulations indicating that, on average, the detected photons in this measurement geometry experience 15 to 20 dynamic scattering events (*3*) and emphasizes that multiple scattering events should never be ignored when analyzing blood flow in the parenchyma.

Knowing the dynamic light scattering regime and the form of the field correlation function *g*_1_(τ) is critical for the correct interpretation of the blood flow and blood flow changes by dynamic light scattering and LSCI methods. One of the major applications of LSCI and LDF for the past 20 years has been mapping and quantifying flow changes during ischemic stroke in animal models (*32*–*35*). The typical models used to analyze LSCI and LDF measurements result in estimates of the relative blood flow in the ischemic core or occluded vessels to be greater than the physiologically expected values (*32*, *34*–*36*). We hypothesized that one of the major reasons for this discrepancy, along with the static scattering effects (*10*) which are also captured by DLSI, is that the incorrect model of the field correlation function has been used to analyze the data. In particular, it has been common to use the SU/MO_*n* = 1_ form of *g*_1_(τ), but the analyses we presented above (Fig. 2B) indicate that it is more accurate to use MU_*n* = 0.5_, at least in the parenchymal regions.

### Ischemic stroke imaging

**Fig. 3:**
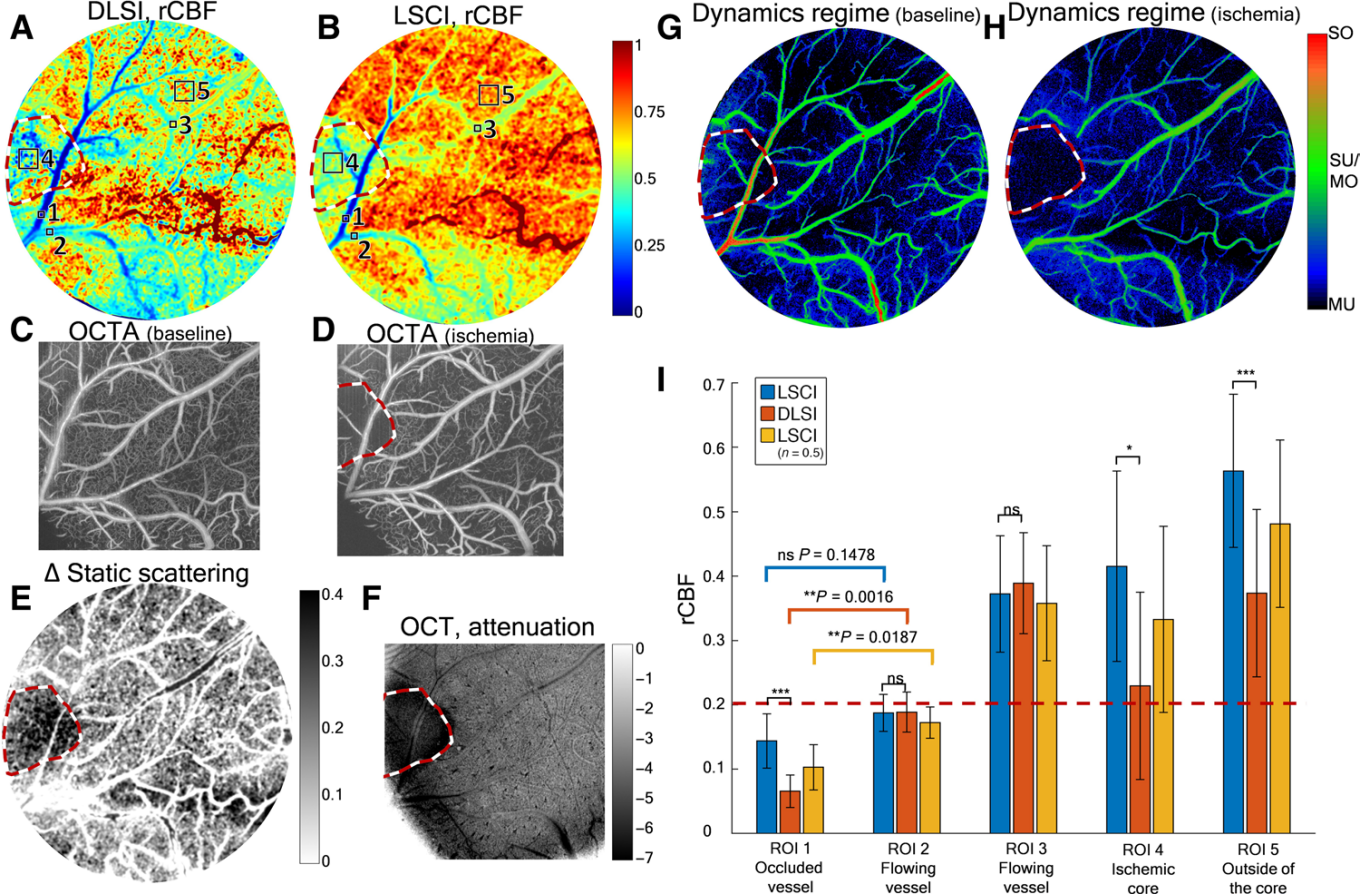
DLSI improves quantitative interpretation of the blood flow. (**A**, **B**, and **I**) Relative cerebral blood flow after the MCA occlusion compared with the baseline flow. The core of the ischemic stroke is approximately indicated with the dashed red-white line in (A) to (H). (A) Map of rCBF_DLSI_ calculated using DLSI. (B) Map of rCBF_LSCI_, calculated using the conventional LSCI model. (**C** and **D**) OCTA images of the corresponding region. (**E**) DLSI estimation of change in static scattering [Δ(1 − ρ)] caused by the stroke. Darker color reflects increase of static scattering, which is particularly evident in the ischemic core. (**F**) OCT signal attenuation, where darker colors also represent increase in static scattering. (**G** and **H**) Dynamic light scattering regimes mapped in baseline and stroke. (I) Mean and SD calculated for ROIs over five animals: occluded artery (ROI 1), large nonoccluded vessels with 70 to 80% (ROI 2) and 50 to 70% (ROI 3) flow reduction, and parenchymal region in the ischemic core of the stroke (ROI 4) and outside of the core (ROI 5). ns, not significant. Exemplary ROI locations shown in (A) and (B). Blue bars correspond to rCBF_LSCI_, orange to rCBF_DLSI_, and yellow to rCBF_LSCI_*n* = 0.5__. The dashed red line shows the physiological stroke threshold of 20% of the baseline flow (*36*).

To test our hypothesis and demonstrate an important application for DLSI, we apply both DLSI and LSCI approaches to measure the relative flow response during a middle cerebral artery (MCA) occlusion–induced stroke in a mouse model (*N* = 5). Associated changes in flow and dynamic scattering parameters are shown in Fig. 3. Relative cerebral blood flow (rCBF) calculated using the conventional LSCI model (rCBF_LSCI_; online methods) results in rCBF_LSCI_ = 0.14 ± 0.04 in the occluded artery [region of interest 1 (ROI 1)], rCBF_LSCI_ = 0.37 ± 0.09 in the parenchyma of the stroke core (ROI 4), and rCBF_LSCI_ = 0.56 ± 0.12 outside of the core (ROI 5; Fig. 2I, blue bars). The respective rCBF_DLSI_, calculated using the model proposed above, is 0.06 ± 0.03, 0.23 ± 0.15, and 0.37 ± 0.13 and is significantly different from rCBF_LSCI_ (*P* < 0.05; Fig. 2I, orange bars) and agrees well with the physiologically expected values (*34*, *36*). Use of the conventional 1/*K*^2^ LSCI model leads to an up to 200% relative error in estimates of rCBF when compared with DLSI, mostly due to the LSCI assumption of SU/MO_*n* = 1_. The error, observed in the occluded arteriole (ROI 1; Fig. 3I), can be associated with the fact that while prestroke DLSI identifies the SU/MO_*n* = 1_ regime in this ROI, the flow reduction during stroke is sufficiently large that the dynamic light scattering regime has changed to MU_*n* = 0.5_ (Fig. 3, G and H). The flow drop is aligned with the areas in the OCT angiogram image (Fig. 2, C and D), in which capillaries are either no longer visible (stroke core) or reduced in number (outside of the core). The core of the stroke is also associated with an increase in static scattering, which is shown by DLSI (Fig. 3E) and OCT signal attenuation (Fig. 3F). As a control, one can see that rCBF in the relatively large nonoccluded (flowing) vessels is almost the same for both techniques: rCBF_LSCI_ = 0.19 ± 0.03 and rCBF_LSCI_ = 0.37 ± 0.09, and rCBF_DLSI_ = 0.19 ± 0.03 and rCBF_DLSI_ = 0.39 ± 0.08 (ROI 2 and ROI 3, correspondingly). This is to be expected since, even at ≈20% of the baseline, flow in these vessels is sufficiently large to not increase static scattering or cause changes in the field correlation function form as happens with the occluded vessel (Fig. 3, E, G, and H). This, however, indicates that techniques requiring assumption of the dynamic scattering regime (i.e., LSCI) might lose the ability to distinguish between flowing and occluded vessels as indicated by the absence of significant difference (*P* > 0.1) between flow in ROI 1 and ROI 2 when conventional LSCI is used. By reanalyzing LSCI data using a simple MU_*n* = 0.5_ model (online methods), we obtain rCBF_LSCI_*n* = 0.5__ = 0.10 ± 0.04, rCBF_LSCI_*n* = 0.5__ = 0.33 ± 0.14, and rCBF_LSCI_*n* = 0.5__ = 0.48 ± 0.13 in ROIs 1, 2, and 3, respectively (see Fig. 2I), which results in much smaller errors, up to 66%, compared with using LSCI with *n* = 1.0. Further theoretical exploration shows that it might be beneficial to generally apply the *n* = 0.5 model in LSCI analysis, as overall it results in lesser error compared with the *n* = 1 model (fig. S9). The only exception would be studies in which only increases in blood flow in large vessels are of interest.

## DISCUSSION

We have introduced the DLSI technique that allows us to measure the speckle intensity temporal autocorrelation function over a wide field of view and accurately estimate the correlation time and static scattering contribution using the most accurate model. We have shown how correlation decay varies in different regions of the brain or, more specifically, in vessels of different sizes. The decay variation is regulated by the amount of photon scattering, the type and magnitude of motion exhibited by the light scattering particles, and by the presence of static scattering. In addition to the practical application of the technique for quantifying blood flow in biomedical applications, with DLSI, we were able to resolve a 30-year-long standing question and determine the best-fitting theoretical model for interpreting laser speckle imaging measurements, which will broadly affect studies using LSCI and laser Doppler blood flowmetry in diverse biomedical disciplines. Contrary to the assumptions made before in LDF, LSCI, and MESI theory, and to our expectations, DLSI showed that the proper *g*_1_(τ) form for flow in smaller vessels and in parenchymal regions corresponds with multiple light scattering from unordered motion. Further, we used DLSI to resolve a discrepancy between the expected physiologically relevant relative blood flow values in ischemic stroke and what has been measured with LDF and LSCI. We have shown that the conventionally applied analysis is unable to distinguish dynamics in the occluded vessel from the vessel with slow blood flow, which might prove critical in potential clinical applications. In the present study, we have focused on the quantitative estimation of the scattering parameters and decorrelation time τ_c_. While it is commonly assumed that τ_c_ is inversely proportional to the flow speed, the exact form of this relationship in LSCI/MESI and LDF is unknown and has been debated for years (*7*, *19*). With DLSI, it will become possible to get an empirical estimate of this relationship and make a step toward measuring absolute blood flow with the relevant techniques.

Despite the fact that DLSI provides invaluable information on the light scattering and particles dynamics, there are a few limitations that have to be resolved to ease the adoption of the technique and widen the range of its applications. The key hardware limitation and the difference from LSCI/MESI imaging systems (*4*, *9*) are related to the frame-rate requirements and utilization of a high-speed camera. In fig. S10, we show how the DLSI frame-rate requirements depend on the decorrelation time τ_c_. A camera with at least 6000 frames/s is needed to fit the dynamics in small vessels and parenchyma, while for vessels with a diameter up to 200 μm, the frame-rate requirement rises to 20,000. An insufficient frame rate will lead to fitting artifacts, such as the sudden change of the dynamics regime observed in the top part of the superior sagittal sinus in Fig. 2A. At the same time, however, it means that when dynamics are slow enough (e.g., τ_c_ > 2 ms), DLSI can be used with a common complementary metal-oxide semiconductor (CMOS) camera. Another limitation is the simplified representation of the noise contribution in the DLSI model, which also exists for LSCI/MESI studies (*9*, *10*, *18*). Further research is required to quantify the noise arising from the high-speed CMOS sensor and light source instabilities (such as mode hopping) and its contribution to the intensity temporal autocorrelation function. In addition, with more DLSI data collected, the number of artifacts can be further reduced by restricting the fitting range of the model parameters based on their expected values. With these limitations taken into account, DLSI will prove useful in applications where the quantitative interpretation of blood flow or measuring the dynamic scattering regime is critical such as in ischemia imaging. In applications where changes in the blood flow are subtle, the error caused by using the wrong model will be much smaller (see fig. S9) and continuous utilization of DLSI will be less beneficial. Nevertheless, in these applications, and in other applications where no changes in dynamics scattering regime are expected or long-term observation is required, we envision that the data-intensive DLSI can be used to calibrate LSCI, and then LSCI is used for efficiently providing long-term quantitative imaging of blood flow dynamics.

## METHODS

### DLSI theory

The DLSI theory models the temporal intensity autocorrelation function and accounts for the dynamics of the scattering particles, static scattering, effects of spatial and temporal speckle averaging, and the form of the field temporal autocorrelation function. In the simplest case, the intensity temporal autocorrelation function *g*_2_(τ) can be related to the electric field correlation function *g*_1_(τ) by the Siegert relation (*8*, *37*)

$$g_{2}(\tau )=1+\beta {|g_{1}(\tau )|}^{2}$$ (2)

where τ is the time lag, and β reflects the effects of the source coherence properties and the spatial (*1*, *16*, *18*) and temporal averaging of the speckle dynamics. When measuring blood flow, *g*_1_(τ) is generally described (*8*) by the form

$$g_{1}(\tau )=\text{exp}(-{\left(\frac{\tau }{\tau_{c}}\right)}^{n})$$ (3)

where τ_c_ is the correlation time constant, and *n* varies depending on the dynamic light scattering regime (*1*–*4*, *13*) (i.e., single versus diffusive light scattering) and the type of motion for the light scattering particles (i.e., ordered versus unordered motion) and takes values of 0.5 (multiple light scattering and unordered motion MU_*n* = 0.5_), 1 (single light scattering and unordered motion/multiple light scattering and ordered motion SU/MO_*n* = 1_), or 2 (single scattering and ordered motion SO_*n* = 2_).

The parameter β is typically assumed to be homogeneous across the field of view. In DLSI, because of the finite exposure time of the camera *T*_exposure_, temporal speckle averaging effects may affect the value of β. Specifically, one can expect that β will decrease when the exposure time is not much much shorter than the correlation time constant τ_c_. While we do not address this question in detail here, to avoid further confusion, we redefine the coherence parameter β as

$$\beta =\beta_{s}\beta_{t}(\tau_{c},T)$$ (4)

where β_s_ captures the decrease in *g*_2_(τ) arising from spatial speckle averaging (*16*) and the partial coherence of the source (*14*, *15*) and β_t_ captures effects arising from temporal averaging. While β_s_ is expected to be constant across the field of view, β_t_ will depend on the sample dynamics at each spatial location and will decrease when the sample dynamics are fast enough such that τ_c_ is no longer much much longer than the camera exposure time. We expect β_t_ to decrease for larger vessels where the correlation time is comparable with the exposure time as can be seen by the *g*_2_(0) values in Fig. 1C and fig. S6. For smaller vessels and parenchymal regions, the β values are expected to be more similar.

#### Measurement noise and insufficient temporal sampling

The experimentally measured *g*_2_(τ) does not always decay to 1 at long lag times but can decay to values larger and smaller than 1 as we observe in fig. S7. When measuring spatial speckle contrast, it is known that the presence of measurement noise and static scattering will result in a constant positive offset term (*9*, *17*, *18*, *38*). However, when measuring *g*_2_(τ) via a temporal intensity autocorrelation measurement, it is known that *g*_2_(τ) decays to 1 even in the presence of static scattering unless spatial ensemble averaging is also performed (*38*–*40*). Thus, our observation of *g*_2_(τ) not decaying to 1 needs further explanation. The biggest factor is the effect of temporal sampling. With sufficient temporal sampling of the speckle fluctuations, the intensity variance will equal the mean intensity squared, that is, <*I*(*t*)*I*(*t* + τ) > = < *I*(*t*)>^2^. This condition of sufficient temporal sampling is met when the camera integration time *T*_exposure_ is much shorter than the correlation time of the speckle intensity τ_c_ and when the length of sampling *T*_total_ is much longer than τ_c_. As we see in fig. S7, the distribution of *g*_2_(τ) at large values of τ becomes more narrowly distributed around a value of 1 when *T*_total_ is increased because we are better meeting the condition of sufficient temporal sampling. However, as *T*_total_ increases, a small positive bias remains in the distribution. This positive bias is a result of measurement noise (specifically the camera readout noise and dark count) reducing the probability of measuring speckles with zero and very small intensities, which skews the temporal intensity distribution such that <*I*(*t*)*I*(*t* + τ)> is greater than <*I*(*t*)>^2^ even at long τ and long *T*. To capture these effects of measurement noise and insufficient temporal sampling in our model, we consider a form for *g*_2_(τ) with a constant offset term *C*

$$g_{2}(\tau )=1+\beta {|g_{1}(\tau )|}^{2}+C$$ (5)

#### Static scattering

As we saw above, in Fig. 1, some vessels appear to have higher values of *g*_2_(τ) at τ = 0 that then decay to lower values at long time lags compared with the surrounding tissue. This behavior is the result of a stronger contribution of static scattering in the parenchyma. While both β_s_ and β_t_ in the parenchyma should be higher or the same compared with vessels, the contribution of static scattering is greater in the parenchyma because of a lower blood volume fraction, leading to a decrease in *g*_2_(0). At longer time lags, however, *g*_2_(τ) in the parenchyma is higher compared with vessels since the speckle dynamics are slower. After the theoretical description in (*9*, *13*, *38*, *40*), we expand the model to include the effects of static scattering

$$g_{2}(\tau )=1+\beta \frac{<I_{f}{>}^{2}}{I^{2}}{|g_{1}(\tau )|}^{2}+2\sqrt{\beta }\frac{I_{c}}{I}\frac{<I_{f}>}{I}|g_{1}(\tau )|+C$$ (6)

where <*I*_f_> is the temporally averaged intensity of the fluctuating component of the speckle intensity, *I*_c_ is the constant component of the static speckle intensity, and *I*_total_ = < *I*_f_ > + *I*_c_ is the total intensity. We denote the fraction of the fluctuating component as $\rho =\frac{<I_{f}>}{I_{\text{total}}}$. Note that when analyzing the temporal correlation function of the speckle intensity, the static scattering does not produce the expected constant offset in the long lag time value of *g*_2_(*u*). As clearly described in (*39*, *40*), this is because the temporal analysis does not have access to the proper intensity statistics of the static speckle intensity *I*_c_, which can only be accessed by analyzing the spatial statistics. A further consequence of this is that *I*_c_ and *I*_total_ are not the true ensemble averaged values of the static and total intensity, and their values will vary across space from speckle to speckle. This variation is evident in the total intensity image shown in fig. S8C, that despite being an average over 4 s still reveals a static speckle pattern. Hence, the proper average total intensity can only be determined from a spatial average of the local speckle pattern. This has an important consequence for temporal LSCI (tLSCI) that has not been described in the literature. While tLSCI provides an accurate measurement of the SD of the temporal speckle fluctuations (σ), it does not provide an accurate measure of the average total intensity, and thus, the speckle contrast *K*_temporal_ = σ/*I*_total_ will not be accurate and will vary unrealistically on the local spatial scale (see fig. S8B). One will have to perform a spatial average of the local speckle intensities to get the proper value for *I*_total_ to use in the calculation of *K*_temporal_. Furthermore, the variation in *I*_c_ and *I*_total_ will modulate effects of the measurement noise and lead to local increases in the constant offset term *C*.

#### *Mixed dynamics in the form for g_1_(*τ*)*

Observing how the quality of fit changes as vessels transition from *SO*_*n* = 2_ to *SU*/*MO*_*n* = 1_ and then to *MU*_*n* = 0.5_ dynamics [see fig. S2, A, D, and E], it is reasonable to expect that the transition between these behaviors is not necessarily sharp. It is highly likely that photons measured from regions with many small/penetrating vessels have experienced multiple scattering from vessels with slow flow exhibiting MU_*n* = 0.5_ dynamics and from vessels with higher flow exhibiting SU/MO_*n* = 1_ dynamics. Limiting the number of dynamic types that can be mixed to two, the field autocorrelation function can be represented as

$$\begin{array}{cc} g_{1}(\tau ) & =\frac{<(E_{1}(t)+E_{2}(t)){\left(E_{1}(t+\tau )+E_{2}(t+\tau )\right)}^{*}{>}_{t}}{<{|E|}^{2}{>}_{t}}\\ & =\frac{<E_{1}(t)E_{1}^{*}(t+\tau ){>}_{t}+<E_{2}(t)E_{2}^{*}(t+\tau ){>}_{t}+<E_{1}(t)E_{2}^{*}(t+\tau ){>}_{t}+<E_{2}(t)E_{1}^{*}(t+\tau ){>}_{t}}{<I_{1}{>}_{t}+<I_{2}{>}_{t}+<E_{1}(t)E_{2}^{*}(t){>}_{t}+<E_{2}(t)E_{1}^{*}(t){>}_{t}}\\ & =\frac{<I_{1}{>}_{t}g_{1,1}(\tau )+<I_{2}{>}_{t}g_{1,2}(\tau )+<E_{1}(t)E_{2}^{*}(t+\tau ){>}_{t}+<E_{2}(t)E_{1}^{*}(t+\tau ){>}_{t}}{<I_{1}{>}_{t}+<I_{2}{>}_{t}+<E_{1}(t)E_{2}^{*}(t){>}_{t}+<E_{2}(t)E_{1}^{*}(t){>}_{t}} \end{array}$$ (7)

where *E*_1_(*t*) indicates the field arising from photon trajectories scattering from particles undergoing the first dynamic type, and *E*_2_(*t*) indicates the field from the second dynamic type. The possible cases of two dynamics mixing are the following:

1) Single scattering ordered SO_*n* = 2_ and multiple scattering ordered MO_*n* = 1_

2) Single scattering ordered SO_*n* = 2_ and single scattering unordered SU_*n* = 1_

3) Single scattering unordered SU_*n* = 1_ and multiple scattering unordered MU_*n* = 0.5_

4) Multiple scattering ordered MO_*n* = 1_ and multiple scattering unordered MU_*n* = 0.5_

5) Single scattering unordered SU_*n* = 1_ and multiple scattering ordered MO_*n* = 1_

6) Single scattering ordered SO_*n* = 2_ and multiple scattering unordered MU_*n* = 0.5_

In each of these cases, *E*_1_(*t*) is not correlated with *E*_2_(*t*). This is because multiple dynamic scattered light never follows the same trajectory as single scattered light and is thus uncorrelated, and because photons that experience scattering from any unordered dynamics are always uncorrelated with other photons. As a result of this, the cross-terms will be equal to zero, and mixed Eq. 7 can be rewritten as

$$\begin{array}{cc} g_{1}(\tau ) & =\frac{<I_{1}{>}_{t}\;g_{1,1}(\tau )+<I_{2}{>}_{t}g_{1,2}(\tau )}{<I_{1}{>}_{t}+<I_{2}{>}_{t}}\\ & =dg_{1,1}(\tau )+(1-d)g_{1,2}(\tau ) \end{array}$$ (8)

where $d=\frac{<I_{1}{>}_{t}}{<I_{1}{>}_{t}+<I_{2}{>}_{t}}$. Since SU_*n* = 1_ and MO_*n* = 1_ have the same form of the correlation function and the case 6 is unlikely to occur, we consider two mixed dynamics models. The first represents mixtures of Mγ_*n* = 0.5_ and SU/MO_*n* = 1_ dynamics

$$\begin{array}{cc} g_{2}(\tau )= & 1+\beta \rho^{2}{\left(d_{\text{MU}}|g_{1}^{n=0.5}(\tau )|+(1-d_{\text{MU}})|g_{1}^{n=1}(\tau )|\right)}^{2}+\\ & 2\sqrt{\beta }(1-\rho )\rho (d_{\text{MU}}|g_{1}^{n=0.5}(\tau )|+(1-d_{\text{MU}})|g_{1}^{n=1}(\tau )|)+C \end{array}$$ (9)

where the *d*_MU_ indicates the fraction of photons with dominant MU_*n* = 0.5_ dynamics. Then, the second represents the mixture of SU/MO_*n* = 1_ and SO_*n* = 2_ dynamics as

$$\begin{array}{cc} g_{2}(\tau )= & 1+\beta \rho^{2}{\left(d_{\text{SO}}|g_{1}^{n=2}(\tau )|+(1-d_{\text{SO}})|g_{1}^{n=1}(\tau )|\right)}^{2}+\\ & 2\sqrt{\beta }(1-\rho )\rho (d_{\text{SO}}|g_{1}^{n=2}(\tau )|+(1-d_{\text{SO}})|g_{1}^{n=1}(\tau )|)+C \end{array}$$ (10)

where *d*_SO_ indicates the fraction of photons with dominant SO_*n* = 2_ dynamics.

These equations can be further generalized into the final DLSI model

$$\begin{array}{cc} g_{2}(\tau )= & 1+\beta \rho^{2}{\left(d|g_{1}^{n=X}(\tau )|+(1-d)|g_{1}^{n=1}(\tau )|\right)}^{2}+\\ & 2\sqrt{\beta }(1-\rho )\rho (d|g_{1}^{n=X}(\tau )|+(1-d)|g_{1}^{n=1}(\tau )|)+C \end{array}$$ (11)

where the *X* value depends on the second dynamic component type: 0.5 for MU_*n* = 0.5_ and 2 for SO_*n* = 2_. The combination of *X* and *d* values defines the dynamic scattering regime that ranges from MU_*n* = 0.5_ to SU/MO_*n* = 1_ and from SU/MO_*n* = 1_ to SO_*n* = 2_. The model can be further expanded to represent a mixture of three dynamics types, but this will make it substantially more complex, is unlikely to be relevant for most physiological applications where *n* = 0.5 and *n* = 1 are the most common, and it will be hard to have confidence that the three dynamics model is better than a two dynamics model.

#### Role of the model components

By setting the parameters to specific values, one can obtain simplified models from the generalized one: Mixed dynamics are removed by setting *d* = 1 and *X* = 0.5 or 2 to get MU_*n* = 0.5_ or SO_*n* = 2_ dynamics or by setting *d* = 0 to get SU/MO_*n* = 1_ dynamics; setting ρ = 1 will remove the static component, and setting *c* = 0 will exclude the constant offset. The model complexity required to fit different regions of the mouse brain is determined using the *F* test and is shown in the fig. S3.

### DLSI imaging and analysis

#### Imaging

A high-speed camera (1280 × 1024 pixels, 991 fps, 5-μm pixel size; Fastec IL5-S, USA) was used to record the backscattered light, through a 5× objective (whole cortex, Mitutoyo, Japan) and VZM 450i imaging lens (stroke experiments, Edmund Optics, USA). A polarizer was placed in front of the objective to increase the contrast. The number of active pixels was reduced to an image stripe of 1280 × 32, and the exposure time *T*_exposure_ was set to 31 μs, allowing the camera to reach the maximum frame rate of 22,881 frames per second. Using this high frame rate makes it possible to accurately fit dynamics with a correlation time as short as 50 μs. We note that slower frame rates can be used when only small vessels and parenchyma are analyzed (fig. S10). Coherent light was delivered to the object using a free-space volume holographic grating (VHG) stabilized laser diode (*15*) (785 nm; LD785-SEV300, Thorlabs, USA) operated at the recommended settings. The light was collimated, passed through the isolator (IO-5-780-VLP, Thorlabs USA), expanded along the long axis of the camera frame with an anamorphic prism pair (PS875-B, Thorlabs, USA), and further expanded by a beam expander (GBE10-B). This resulted in an ≈10 × 2–mm, 200-mW power beam spot at the surface of the brain, which was then aligned with the camera’s field of view as shown in the bottom right part of Fig. 1A. The size of the speckle on the camera was adjusted by altering the pupil diameter of an iris in the detection path to achieve a speckle-to-pixel size ratio of approximately 2. To scan the whole field of view, the stage with the animal (LTS150, Thorlabs, USA) was translated in the *Y* direction such that sequential image stripes had an overlap of 10 pixels to permit registration of sequential stripes. Each stripe was recorded for 4 s (whole cortex) or 2 s (stroke), resulting in 91,544 or 45,772 speckle images per stripe.

#### Intensity correlation

The image stripes were stitched together by spatial coregistration of the τ_c_ images, and the overlap was removed. The camera black level was evaluated and subtracted from the intensity before the analysis. The resulting speckle images were processed to obtain the speckle intensity temporal autocorrelation function *g*_2_(τ) (*38*–*41*)

$$g_{2}(\tau )=\frac{\langle I(t)I(t+\tau )\rangle }{{\langle I(t)\rangle }^{2}}$$ (12)

where *I* is the intensity recorded at a specific pixel, *t* is a time corresponding to the current frame, and τ is the time lag value. Angle brackets denote averaging across the observation time *T*_total_ = 4 s. Depending on the experiment, the time lags τ ranged from either 0 to 8.8 ms for whole-cortex imaging or from 0 to 17.2 ms for stroke imaging. Longer time lags were used for stroke imaging because of the slower blood flow and, thus, slower decorrelation.

#### Model fitting

The calculated intensity temporal autocorrelation function was fit with the generalized Eq. 11 (Fig. 2 and fig. S3, G to I) and its simplified versions (Eqs. 2, 5, and 6 using a nonlinear least squares fitting algorithm in MATLAB 2018b function “fit” with default parameters. To analyze model parameters (figs. S2 and S3), the parameter fitting ranges were constrained to 0..1 for β, ρ, *d*_MU_, and *d*_SO_; −1..1 for *C*; and 0..8.8 or 0..17.2 ms for τ_c_ for whole-cortex and stroke imaging, respectively. Initial conditions for the correlation time constant τ_c_ini__ were identified by finding the time lag at which *g*_2_(τ) crosses the value $1+\frac{g_{2}(0)-1}{e^{2}}$ (*42*). To avoid fitting artifacts, the results shown in Fig. 2 were calculated with an adaptive constraint of the fit range for parameters β and ρ. More specifically, pixels were fit in the order corresponding to descending values of τ_c_ini__. As we observed that β and ρ tended to get smaller and larger, respectively, with decreasing τ_c_, after the first 30% of pixels are fit, the upper boundary for β and lower boundary for ρ are defined as <β > + 2σ_β_ and <ρ > − 2σ_ρ_ correspondingly, where <> and σ are mean and SD of the fit parameter over the last 30% of fitted pixels and are recalculated for each next pixel. This constraint helps to avoid fitting artifacts that resulted in small values of β in pixels with longer correlation times (parenchyma) and small values of ρ in pixels with shorter correlation times (large vessels). The value *g*_2_(0) was excluded from the fitting data for all pixels, due to the uncorrelated acquisition noise strongly affecting the zero lag correlation. The “tail” of the intensity correlation curve, starting from the point where the correlation drops below 10% of the initial value, was also excluded from the fitting data, except for the cases where the total number of points was less than 5 (large vessels with fast decorrelation). In those cases, 5 points, starting from the first lag, were used.

#### Model testing with the F test

To determine the best model required to fit different regions of the brain, the *F* test (*43*) was performed (fig. S3, C, F, and I). We started with the simplest model with the fewest parameters and selected increasingly more complex models when the *F* test proved significant with *P* ≤ 0.05.

#### Blood flow

The relative blood flow changes measured with DLSI were calculated as

$$\text{rCB}F_{\text{DLSI}}=\frac{\tau_{c_{\text{baseline}}}}{\tau_{c_{\text{stroke}}}}$$ (13)

where τ_c_baseline__ and τ_c_stroke__ are the correlation times obtained by fitting the chosen model to the corresponding experimental data.

The code for calculating and fitting the intensity temporal autocorrelation function was written in MATLAB and executed using the graphical processing unit to achieve a reasonable performance. The code is available in the BU Neurophotonics GitHub repository (*44*).

### Laser speckle contrast imaging

To obtain laser speckle contrast images from the same dataset, sequences of 114 speckle images were averaged to provide a close equivalent to the image captured with a 5-ms exposure time. The speckle contrast was calculated and converted to the blood flow index (BFI) according to the commonly used simplified model (*35*, *45*–*48*)

$$\text{BFI}=\frac{1}{K^{2}}=\frac{\sigma^{2}}{{\langle I\rangle }^{2}}$$ (14)

where *K* is contrast, σ and 〈*I*〉 are the SD and the mean of the intensity (i.e., the pixel values) over either a 5 × 5 neighborhood [spatial contrast (*18*); see Fig. 2] or 25 frames [temporal contrast (*49*); see fig. S8]. This resulted in 803 (spatial contrast) or 32 (temporal contrast) images that were then averaged, providing the contrast information averaged over the entire observation period *T*_total_ = 4 s to obtain a single spatial/temporal speckle contrast image.

#### Modified model

To analyze contrast under the assumption of multiple scattering and unordered dynamics (MU_*n* = 0.5_), we find τ_c_ by solving the following equation

$$\begin{array}{cc} K^{2}= & (\frac{\tau_{c}}{T}(1+2\text{exp}(-2\sqrt{T/\tau_{c}}))+3\frac{\tau_{c}\sqrt{\tau_{c}}}{T\sqrt{T}}\text{exp}(-2\sqrt{T/\tau_{c}})+\\ & \frac{3}{2}\frac{\tau_{c}^{2}}{T^{2}}(\text{exp}(-2\sqrt{T/\tau_{c}})-1)) \end{array}$$ (15)

where *T* is exposure and *K* is spatial contrast calculated as stated above. Details of the derivation of Eq. 15 can be found in the Supplementary Materials.

#### Relative blood flow

Relative blood flow changes during stroke were calculated from the contrast data as

$$\text{rCB}F_{\text{LSCI}}=\frac{\text{BF}I_{\text{stroke}}}{\text{BF}I_{\text{baseline}}}=\frac{K_{\text{baseline}}}{K_{\text{stroke}}}$$ (16)

where *K*_baseline_ and *K*_stroke_ are the spatial contrast calculated according to Eq. 14 during the baseline and ischemic stroke, respectively.

### Optical coherence tomography angiography (OCTA)

For the stroke experiment, optical coherence tomography angiography was performed (*50*). A spectral-domain optical coherence tomography (OCT) system (1310-nm center wavelength, bandwidth of 170 nm; Thorlabs Telesto III) was used for imaging of the microvasculature of the cerebral cortex. The axial resolution of the system in the air was 4.6 mm. Given the light refractive index of 1.35 for brain tissue, the axial resolution in the brain was 3.5 mm. The imaging speed was 76,000 A-scan/s. A 5× objective [5× Mitutoyo Plan Apochromat objective, 0.14 numerical aperture] was used in this study. The microvascular angiogram was constructed by the subtraction of repeat B-scans (*50*). OCT data were acquired over an area spanning 500 × 500 pixels equivalent to 2000 × 2000 × 1000 μm^3^. Each angiogram volume acquisition took ≈9 s. Ten volumes were acquired and then averaged to increase the signal-to-noise ratio. Angiogram images were presented as the maximum intensity projection over a depth range of 1 to 250 μm from the brain surface.

### Animal preparation

All animal procedures were approved by the Boston University Institutional Animal Care and Use Committee and were conducted following the *Guide for the Care and Use of Laboratory Animals*. Two experiments were designed: one for the imaging of the whole cortex and another one for monitoring of the blood flow change during the stroke.

#### Whole-cortex imaging

C57Bl6 mice were anesthetized with isoflurane [3% induction, 1 to 1.5% maintenance, in oxygen (1 liter/min)] during surgery and imaging sessions. After removal of the scalp, the skull was removed to fit the placement of the crystal skull (*51*). The glass then was sealed with dental acrylic, and the animal was recovered for 3 weeks before the imaging session. During surgery and imaging, heart rate and oxygen saturation were noninvasively monitored (Mouse Stat Jr., Kent Scientific), and all noted measurements were within the expected physiological range.

#### Stroke imaging

*N* = 5 animals (approximately 15-week-old male C57BL/6 mice) were used for the stroke experiments. For each animal, we followed the same procedure. The animal was anesthetized with isoflurane (2 to 3% induction, 1 to 2% maintenance, in 60% nitrogen, 40% oxygen mixture). After a midline skin incision and removal of scalp and periosteum, a custom-made aluminum bar was glued over the left half of the skull, for fixation of the head. Then, approaching between the right lateral epicanthus and external auditory meatus, the temporalis muscle was dissected to reveal the squamous portion of the temporal bone. The temporal bone over the distal MCA, 1 mm above the zygomatic arch, was drilled, and a craniotomy of 2-mm diameter was opened, with dura intact. To prevent overheating, extended drilling of the same area for more than 2 to 3 s was avoided, and the skull was cooled with artificial cerebrospinal fluid (aCSF) at room temperature throughout the drilling process. The exposed MCA and surrounding cortex were flushed with warm (at 37°C) aCSF and then was covered with aCSF-soaked gauze for protection. Next, another frontoparietal craniotomy was opened (4 mm in diameter) to visualize the MCA-supplied cortex. The cortex was then covered with 0.7% agarose solution in aCSF, followed by a 5-mm glass coverslip. The window was sealed with dental cement. The animal was then placed under the imaging system. We waited for at least 30 min for stabilization of the cerebral blood flow until we started the baseline imaging. During the entire length of the surgical procedure, the animal was heated by a homeothermic blanket with rectal-probe feedback to maintain the temperature at 37°C. Arterial oxygen saturation and heart rate were monitored noninvasively with a paw probe (MouseSTAT Jr., Kent Scientific Instruments).

We used a ferric chloride–induced MCA thrombosis model, as previously described (*34*). After the acquisition of baseline imaging data, a piece of filter paper (0.3 × 0.5 mm, Whatman No.1) soaked in 30% ferric chloride (FeCl3; the solution in isotonic saline) was placed over the intact dura matter along the trace of the MCA right after the zygomatic arch. This concentration of FeCl3 was preferred to prevent spontaneous recanalization of MCA, based on previous experience (*34*). The paper was kept in place for 10 min, and then the flow in pial MCA branches was checked with LSCI. Since residual flow was detected, another piece of soaked paper was reapplied for 5 min, and repeated LSCI confirmed distal middle cerebral artery (dMCA) occlusion. The FeCl3-soaked paper was then removed, and the dMCA trunk area was washed with warm aCSF. Before the DLSI analysis, the ischemic core was identified on the basis of the OCTA and OCT signal attenuation images [see Fig. 3)] as the area with almost no capillary flow and strong signal attenuation (*52*, *53*).

### Statistical analyses

Paired *t* test was applied to compare between LSCI and DLSI measurements and to perform the comparison between ROIs. *P* values above 0.05 are reported as not significant. Results were expressed as means ± SD unless otherwise indicated.

## SUPPLEMENTARY MATERIALS

Supplementary material for this article is available at http://advances.sciencemag.org/cgi/content/full/6/45/eabc4628/DC1

## Appendix

View/request a protocol for this paper from *Bio-protocol*.
